# The Influence of the COVID-19 Pandemic on Small Business Support for Youth Physical Activity Opportunities in Urban, Low-Income, Predominantly Black Neighborhoods

**DOI:** 10.1007/s40615-024-02185-9

**Published:** 2024-10-01

**Authors:** Richard R. Suminski, Kristin Kelly, Cora J. Firkin

**Affiliations:** Department of Health Behavior and Nutrition Sciences, University of Delaware, Newark, DE 19716 USA

**Keywords:** COVID-19, Pandemic, Physical activity, Black youth

## Abstract

**Background:**

A relatively low percentage of Black youth meet physical activity (PA) guidelines. An important resource for helping Black youth be physically active is the availability of quality youth physical activity opportunities (YPAO) which manifest, in part, due to support from small businesses. The coronavirus disease 2019 (COVID-19) pandemic was a devastating negative societal event that disproportionately burdened the Black community. How the pandemic influenced the relationship between small businesses and YPAOs in this community is vital for understanding the promotion of PA in this high-risk population going forward.

**Purpose:**

To describe small business support for YPAO before and after 2 years of exposure to the COVID-19 pandemic.

**Methods:**

In-person interviews were conducted June through August 2019 (pre-COVID) and 2021 (post-COVID) with owners and managers at small businesses in 20 urban, low-income, predominantly Black neighborhoods.

**Results:**

The number of YPAOs supported was significantly greater pre-COVID (*n* = 104) versus post-COVID (*n* = 52) (*t* = 4.6; *p* < .001). From pre-COVID to post-COVID, the types of YPAOs supported by businesses shifted from a diverse mix to mostly (96%) outdoor, community sports teams. Businesses were more likely to provide goods and services (*p* = .02) for YPAOs pre-COVID than post-COVID. The major reason for not supporting YPAOs pre-COVID was “not being asked for support” then “not being able to locate YPAOs to support” post-COVID.

**Conclusion:**

Exposure to COVID-19 is associated with significant changes in how small businesses support YPAOs. The results are useful for informing strategies and public policies aimed at promoting YPAO support through prominent negative societal events.

## Introduction

Regular physical activity (PA) participation is associated with health benefits for youth under 18 years of age, including reductions in risk for obesity, type 2 diabetes, cardiovascular disease, and specific cancers [1–4]. Nevertheless, a considerable proportion of youth in the U.S. do not attain adequate PA levels: 84% of teens do not meet both aerobic and muscle-strengthening guidelines and 7.6% of children do not participate in any aerobic or muscle-strengthening PA [5]. Racial disparities in PA participation are well-documented, with the 2021 Youth Risk Behavior Surveillance Survey reporting only 10.8% of Black youth meet PA guidelines compared to18.6% of White youth [5]. This is not a new trend; data from 1995 showed a significant racial disparity in PA, with Black youth participating in less PA than their White peers [6]. These persistent disparities likely contribute to the disproportionately high prevalence rates of health conditions among Black youth, with 8% classified as severely obese compared to 3.9% of White youth and the incidence of type 2 diabetes in Black youth is five times higher than their White counterparts [7–9].

The factors contributing to these racial disparities in PA participation are multifaceted, extending beyond individual behavior to encompass a range of social and environmental determinants. Among these, environmental determinants such as access to parks and recreational facilities, the quality of the built environment, and public transportation infrastructure have emerged as some of the most influential [10–12]. For youth, an impactful environmental determinant of PA is the availability and quality of youth physical activity opportunities (YPAO), which encompass are programs and places with amenities and resources to promote youth PA (e.g., sports leagues, playgrounds) [13–15]. Significant associations have been found between youth PA and access to affordable and local (e.g., closer to home) YPAOs, and lack of YPAOs has been cited by youth as a major barrier to being active [16–18]. Yet, communities with a higher proportion of Black Americans are associated with fewer YPAO settings, such as sports facilities, parks, and public pools [19]. In addition to availability, the quality of YPAOs (e.g., conditions of facilities) also influences youth PA [20, 21]. A 2024 review highlighted inequities in the quality of neighborhood parks, open spaces, and sports facilities across socioeconomic statuses [22]. Considering the intersection between socioeconomic status and race, YPAOs vary between neighborhoods with h economically disadvantaged and predominantly Black neighborhoods having significantly fewer YPAOs of lower quality than affluent, predominantly White neighborhoods [21, 23–26]. These disparities, compounded by the costs associated with YPAO participation, have been identified as major barriers preventing low-income, Black youth from becoming physically active and maintaining PA participation [27, 28]. Addressing these gaps requires strategies to promote equitable access to quality YPAO, particularly within underserved neighborhoods, to help mitigate health disparities linked to youth inactivity.

Small businesses, defined as having fewer than 500 employees, account for 99.9% (32 million) of all businesses operating in the U.S. Total revenues typically exceed one trillion dollars annually, and over half a million new small businesses start each month [29]. They represent a potential avenue for improving YPAO access and quality. Small businesses have been shown to bolster local community health initiatives, including YPAOs, through sponsorships and by contributing goods and services [15, 30–33]. Such local support for YPAOs can enhance the access and quality of YPAOs, particularly in urban, low-income, racially diverse neighborhoods, ultimately contributing to youth PA participation [34]. In turn, small businesses benefit from expanded and more efficient marketing, increased consumer support, and additional revenue [35–37]. This reciprocal relationship between small businesses and YPAOs can be leveraged to help design healthier, more active neighborhoods.

The SARS-CoV-2 virus, responsible for the emergence of the coronavirus disease 2019 (COVID-19) pandemic, marked a global crisis that substantially affected health and well-being. In the U.S., the pandemic began in March 2020, with over six million hospitalizations and 1.1 million COVID-19-related deaths as of 2023 [38]. By October 2020, roughly 11 million (over one out of ten) Americans were unemployed, with more than half of unemployment due to business closures due to COVID-19 transmission reduction measures or the economic impact of lost commerce [39–41]. Unfortunately, small businesses, particularly those located in Black communities, faced the greatest loss of business during the pandemic due to their sensitivity to greater and longer-term COVID-19 consequences [39].

Since 2020, additional health impacts have extended beyond the SARS-CoV-2 infection and symptoms to comprise indirect effects on multi-level factors associated with PA. The COVID-19-related changes to public policies, including quarantines, resulted in the cessation of a diverse set of YPAOs, including the closure of several organizations, including gyms, sports leagues, public recreational spaces, and schools [42, 43]. In schools, for example, youth had limited opportunities to engage in physical education, recess, after-school PA programs, and extracurricular sports teams [44]. These negative effects exacerbated existing disparities in access to YPAOs across communities, contributing to a widening gap in PA participation among racially diverse youth [39, 42]. Beyond the impact of policy changes on YPAOs, it is possible that they experienced additional difficulty operating during the pandemic due to a loss of support from small businesses. However, empirical evidence on this contention is lacking, but needed to understand how to improve the resilience and sustainability of YPAOs when faced with negative societal events such as the COVID-19 pandemic.

Although the pandemic did not allow us to conduct our original study (implement and evaluate an intervention to promote small business involvement with YPAOs), it did create an opportunity to examine its impact on YPAO-small business relationships. Thus, the present study sought to describe small business support for YPAOs in urban, low-income, predominantly Black neighborhoods prior to and after 2 years of exposure to the COVID-19 pandemic. More specifically, this study attempted to determine how the pandemic changed small business support for YPAOs in terms of frequency and types of support, the extent small business and owner characteristics relate to YPAO support pre-and post-COVID, and the reasons for supporting or not supporting YPAOs pre- and post-COVID.

## Methods

### Study Areas

A multi-step process involving reviews of land-use maps and census tract data was used to define neighborhoods in an urbanized area (population > 50,000) of New Castle, County, DE [45]. Twenty of these neighborhoods met the inclusion criteria requiring > 75% of residents to self-identify as Black, median household yearly incomes in the lower third of all neighborhoods in the urbanized area, and land-use mixes of > 30% residential and > 10% retail/commercial. The 20 neighborhoods had the following characteristics [Mean (standard deviation)]—total population 3477 (1003), percent of total population Black 89.9% (7.4%), median household yearly income $30,646 ($8,081), percent of total population 5–18 years of age 29.7% (5.2%), and percent of land commercial/retail 12.8% (2.7).

### Procedures

Outcome measures were obtained June through August 2019 (pre-COVID) and June through August 2021 (post-COVID)] on separate samples of randomly selected small businesses within the study neighborhoods. Immediately prior to the 2019 pre-COVID assessments, 1213 small businesses had a current license to conduct business in at least one of the study areas. In-person visits were made to all 1213 businesses to confirm additional inclusion criteria, including if the businesses had a physical location, was accessible to the public, safe to approach, and in business. Once a business was identified as fully eligible, a trained interviewer completed a survey with the owner. If the owner was not available, a supervisor or manager completed the survey if they indicated having sufficient knowledge of the business plans and policies to answer the survey questions. Eligible businesses were visited a maximum of three times on different days and times of the day. If a survey was not completed after three attempts, the business was excluded.

Post-COVID, 695 small businesses that did not complete a pre-COVID survey were currently licensed to operate in at least one of the study areas. The same pre-COVID procedures were followed to complete surveys with a second sample of fully eligible businesses. The research protocol was approved by the University of Delaware’s Institutional Review Board for the protection of human subjects in research (#1,162,247–10).

### Measures

#### Small Business Policy (SBP) Survey

The SBP survey is a reliable (*κ* = 0.83) survey developed to capture the presence and development of small business support for YPAOs [15, 29, 34]. The SBP includes questions about business (e.g., marketing budget) and owner (e.g., age) characteristics, current involvement with community initiatives, and support for YPAOs, including the cost, location, and reason for each YPAO supported. Questions were added to the post-COVID survey to garner information on how the pandemic impacted the business (e.g., closed, reduced hours, revenue changes).

### Statistical Analysis

Data are presented as means ± standard deviations or percentages when applicable. Continuous variables (# of YPAOs supported, budget amounts, # employees and locations, and owners’ ages) compared between pre- and post-COVID groups of businesses were void of extreme outliers and normally distributed according to Shapiro–Wilk tests. Thus, a parametric, independent samples *t*-test was used to compare the continuous variables between pre- and post-COVID samples. Chi-Square tests were performed when comparing categorical variables (YPAO support offered, types of YPAOs supported, support type, reasons for support/non-support, and business/owner characteristics) between pre- and post-COVID groups. Alpha was set a priori at 0.05 and all analyses were performed with the SPSS statistical software package (IBM Corp. Released 2020. IBM SPSS Statistics for Windows, Version 27.0. Armonk, NY: IBM Corp).

## Results

### Sample

Of the 1213 small businesses visited pre-COVID, 350 (28.9%) did not meet the inclusion criteria. Pre-COVID surveys were completed at 223 (25.9%) of the 863 fully eligible businesses, with 507 (58.7%) not completing a survey after three attempts and 133 (15.4%) refusing to participate. Of the 695 small businesses visited post-COVID, 140 (20.1%) did not meet the inclusion criteria. Post-COVID surveys were completed at 191 (34.4%) of the 555 fully eligible businesses, with 301 (54.2%) not completing a survey after three attempts and 63 (11.4%) refusing to participate. Most surveys (60.4% pre-COVID and 64.2% post-COVID) were completed by the business owner. Among the businesses surveyed post-COVID, 34.7% closed temporarily during COVID, 42.3% slowed down (reduced hours, customers), 2.2% were positively impacted, and 7.7% were not impacted.

### Change in YPAO Support

The proportion of small businesses supporting YPAOs did not differ significantly [*Χ*^*2*^(1,413) = 2.23; *p* = 0.14] between pre-COVID (33.6%, *n* = 75) and post-COVID (26.8%, *n* = 51). However, the number of YPAOs supported was significantly greater (*t* = 4.6; *p* < 0.001) pre-COVID (*n* = 104 YPAOs/75 businesses or 1.4 YPAOs/business) versus post-COVID (*n* = 52 YPAOs/51 businesses or 1.0 YPAOs/business).

Descriptions of small businesses supporting YPAOs are provided in Table 1. The types of YPAOs supported by small businesses were more diverse pre-COVID than post-COVID. Support for community sports teams post-COVID (96.0%) was significantly greater [*Χ*^2^(1125) = 20.41; *p* < 0.001] than pre-COVID (53.3%). Statistical comparisons could not be made for the other types of YPAOs due to insufficient numbers of businesses supporting these types post-COVID. In terms of type of support, donating money [*Χ*^2^(1125) = 0.46; *p* = 0.50] and volunteering time [*Χ*^2^(1125) = 2.08; *p* = 0.15] were not significantly different pre-COVID and post-COVID. However, supporting YPAOs by providing goods and services was significantly greater [*Χ*^2^(1125) = 5.19; *p* = 0.02] pre-COVID (28.6%) versus post-COVID (12.0%). Reasons for supporting YPAOs were similar pre-COVID and post-COVID, with “good will” being the most prominent reason [pre: 50.5%; post: 63.0%; *Χ*^2^(1125) = 3.21; *p* = 0.07] followed by having an affiliation with a YPAO [pre: 32.0%; post: 22.4%; *Χ*^2^(1125) = 1.07; *p* = 0.31]. Regardless of the assessment point, very few businesses supported YPAOs because they were asked or for marketing purposes.

**Table 1:** Descriptions of small business support for YPAOs

	Pre (*n* = 75 businesses)	Post (*n* = 51 businesses)
**Types of YPAO (%)**		
Community team sports leagues	53.3**	96.0
School athletics	14.3	2.0
Community non-profit event or program	24.7	2.0
For profit program	4.8	0
Faith-based organization sports program	2.9	0
**Type of support (%)**		
Goods and services	28.6*	12.0
Monetary ($)	60.0	66.0
Time	11.4	22.0
**Reasons for support (%)**		
Good will	50.5	63.0
Affiliation	32.0	23.4
Solicited (asked for support)	8.7	6.8
Marketing purposes	8.7	6.8

^*^p < .05

^**^p < .001

Reasons for not supporting YPAOs given by small businesses not supporting YPAOs are shown in Table 2. Relatively small and similar percentages of businesses pre-COVID (12.2%) and post-COVID (15.9%) indicated lack of finances as being a reason for not supporting YPAOs [*Χ*^2^(1258) = 3.2; *p* = 0.58]. Businesses having practices/policies that did not include provisions to support YPAOs were significantly more prevalent pre-COVID than post-COVID [*Χ*^2^(1258) = 13.3; *p* < 0.001]. Nearly 40% of businesses not supporting YPAOs pre-COVID were not asked to support YPAOs, whereas only 6.1% post-COVID indicated not being asked for support [*Χ*^2^(1258) = 43.3; *p* < 0.001]. Further, 6.5% of businesses pre-COVID compared to 43.9% of businesses post-COVID could not find YPAOs to support [*Χ*^2^(1258) = 45.4; *p* < 0.001]. Finally, having to close during the pandemic was a reason for not supporting YPAOs given by 13.6% of the businesses post-COVID. None of the businesses pre-COVID gave this as a reason.

**Table 2:** Reasons for not supporting YPAOs among businesses not currently supporting YPAOs pre- and post-COVID (all values are %)

	Pre (*n* = 148)	Post (*n* = 139)
Unsolicited (not asked for support)	39.6**	6.1
Financial	12.2	15.9
Could not find YPAOs to support	6.5**	43.9
Not a business practice/policy	38.9**	20.5
Business was closed	0	13.6

^**^p < .001

### Small Business and Owner Characteristics

Characteristics of businesses supporting YPAOs by survey year are presented in Table 3. Businesses supporting YPAOs pre-COVID were nearly 13 times more likely than business supporting YPAOs post-COVID to also support non-YPAO community initiatives [*Χ*^2^(1126) = 57.1; *p* < 0.001]. Pre-COVID businesses supporting YPAOs also had greater annual budgets for community initiatives (*t* = 3.6; *p* < 0.001; 95% CI 1111.6, 4067.7) and advertising (*t* = 2.5; *p* = 0.02; 95% CI 3154.1, 27,966.5), more employees (*t* = 2.2; *p* = 0.04; 95% CI 0.02, 11.5), and had been in business longer (*t* = 2.7; *p* = 0.01; 95% CI 1.6, 11.7) than post-COVID businesses supporting YPAOs. The only business characteristic that did not differ significantly between the pre-COVID and post-COVID groups was the number of locations (*t* = 2.0; *p* = 0.62; 95% CI − 0.01, 1.20).

**Table 3:** Characteristics of small businesses supporting and not supporting YPAO pre- and post-COVID

	Supporters		Non-supporters	
	Pre (*n* = 75)	Post (*n* = 51)	Pre (*n* = 148)	Post (*n* = 139)
Support non-YPAO community initiatives % yes	76.0***	5.9	51.4***	12.9
Annual budget $ for community initiatives [M(SD)]	3358 (3359)***	768 (495)	7931 (2231)**	657 (548)
Total employees	16.1 (23.1)*	10.3 (8.8)	13.8 (30.5)*	8.6 (7.5)
Years in business	19.8 (20.1)*	13.1 (6.6)	16.6 (17.7)*	13.3 (9.1)
Number of locations	1.9 (2.5)	1.3 (0.9)	1.9 (3.4)	1.3 (1.1)
Annual advertising budget ($)	17,080 (38,170)*	1519 (1726)	25,902 (10,115)*	678 (1,010)

^*^p < 0.05

^**^p < 0.005

^***^p < 0.001

Characteristics of businesses not supporting YPAOs also differed between pre-COVID and post-COVID (Table 3). Businesses not supporting YPAOs pre-COVID were about four times more likely to support non-YPAO community initiatives than businesses not supporting YPAOs post-COVID [*Χ*^2^(1280) = 44.8; *p* < 0.001]. The businesses not supporting YPAOs pre-COVID versus those not supporting YPAOs post-COVID had greater annual budgets for community initiatives (*t* = 3.4; *p* = 0.001;95% CI 3515.2, 8064.0) and advertising (*t* = 2.4; *p* = 0.03;95% CI 908.1, 51,355.5), more employees (*t* = 2.2; *p* = 0.04;95% CI 0.02, 10.4), and had been in business longer (*t* = 2.2; *p* = 0.04; 95% CI 0.02, 6.6). The number of locations did not differ significantly between pre-COVID and post-COVID businesses not supporting YPAOs (*t* = 1.8; *p* = 0.07; 95% CI − 0.04, 1.1).

Owners of business supporting YPAOs post-COVID were about three times more likely than owners of businesses supporting YPAOs pre-COVID to live in their businesses’ zip code [*Χ*^2^(1126) = 26.3; *p* < 0.001] (Table 4). Pre-COVID and post-COVID owners were similar with regards to age (*t* = 1.9; *p* = 0.06; 95% CI − 0.14, 10.4), sex [*Χ*^2^(1126) ≤ 0.01; *p* = 0.91], having a sports background [*Χ*^2^(1,126) ≤ 0.01; *p* = 0.98], having children [*Χ*^2^(1126) = 1.81; *p* = 0.18], and believing a small business should support YPAOs [*Χ*^2^(1126) ≤ 0.01; *p* = 0.99].

**Table 4:** Owner characteristics of small business supporting and not supporting YPAO pre- and post-COVID

	Supporters		Non-supporters	
	Pre (*n* = 75)	Post (*n* = 51)	Pre (*n* = 148)	Post (*n* = 139)
	Mean	Mean	Mean or %	Mean
Age years [M(SD]	45.1 (13.4)	40.0 (13.6)	45.0 (14.7)**	37.4 (14.1)
Sex % male	58.7	56.0	63.3	62.4
Sports background % yes	74.7	76.5	56.8	65.7
Have children % yes	85.3	74.0	69.6	79.7
Small businesses should support % yes	98.6	100	89.6*	97.7
Live in business’s zip code % yes	21.3**	68.6	23.3*	38.8

^*^p < 0.01

^**^p < 0.001

Also given in Table 4 are owner characteristics of small businesses not supporting YPAOs. Owners of businesses not supporting YPAOs post-COVID were approximately 7.5 years younger (*t* = 4.1; *p* ≤ 0.001; 95% CI 3.0, 11.3), more likely to live in their businesses’ zip code [*Χ*^2^(1280) = 7.02; *p* = 0.01], and believe small businesses should support YPAOs [*Χ*^2^(1280) = 6.30; *p* = 0.01] than owners of businesses not supporting YPAOs pre-COVID. Pre-COVID and post-COVID business owners did not differ on sex [*Χ*^2^(1280) ≤ 0.01; *p* = 0.98], having a sports background [*Χ*^2^(1280) = 1.99; *p* = 0.16], or having children [*Χ*^2^(1280) = 3.24; *p* = 0.07].

## Discussion

The purpose of this study was to describe small business support for YPAOs in urban, low-income, predominantly Black neighborhoods prior to and after 2 years of exposure to the COVID-19 pandemic. Several aspects associated with small business support or non-support for YPAO differed between pre-COVID and post-COVID such as the kinds of support provided, availability of YPAOs to support, and characteristics of businesses and their owners. Further, because of the pandemic, many small businesses reduced their hours of operation or closed temporarily or permanently. Some of these identified changes undoubtedly contributed to the lower number of YPAOs supported by businesses post-COVID. The findings provide insight into how a major health crisis influences relationships between small businesses and YPAOs.

Although the proportion of small businesses supporting YPAOs did not differ significantly between pre-COVID and post-COVID, the number of YPAOs supported was significantly lower after COVID exposure. Pre-COVID 104 YPAOs were supported by 75 businesses (1.4 YPAOs/business), whereas post-COVID 52 YPAOs were supported by 51 businesses (1.0 YPAO/business). This decrease is consistent with the overall reduction in businesses’ sponsorship activities noted the COVID-19 pandemic [46]. While reductions in sponsorship activities by businesses have been attributed to economic conditions (e.g., inflationary economy), the results of the current study suggest that economic/financial concerns do not have a significant influence on small business support for YPAOs during major public health crises [47]. This finding could be important for maintaining small business-YPAO relationships (especially during negative societal events) given that most (> 60%) of small business support for YPAOs is financial and that only a small percentage (~ 16%) indicated they did not support YPAOs because of financial reasons. The primary reason small businesses reduced their support for YPAOs was due most likely to a lack of YPAOs to support, which corresponds with the closing of most YPAOs during the pandemic. While this was unavoidable during COVID-19, plans should be developed to help YPAOs counter the negative effects of the next pandemic. This could, for example, involve allocating a portion of small business support to activities YPAOs have to endure to continue safe operations during and after a pandemic (e.g., covering the cost of viral transmission mitigation).

In addition to quantity, a shift in the types of YPAOs supported by small businesses was evident. Pre-COVID, small businesses supported a somewhat diverse group of YPAOs, with approximately half (53%) supporting community sports leagues followed by community non-profit events or programs (25%), school athletics (14%), for-profit programs (5%), and faith-based sports programs (3%). The proportion of businesses supporting YPAOs shifted primarily to community sports leagues (96%) the bulk (92%) of which were conducted outdoors (e.g., little league baseball/softball). While indoor and outdoor YPAOs were affected by pandemic-related regulations, indoor YPAOs had additional obligations that severely limited their availability more so than outdoor YPAOs [48]. The absence of support for YPAOs other than community sports leagues, which decreased from 47% before to 4% after the pandemic, is also notable. Of particular concern in Black communities is the observed drop in the proportion of faith-based sports programs supported by businesses (3 to 0%) in the same neighborhoods. Faith-based organizations have historically been key for providing PA opportunities in the Black community, with emerging evidence supporting the impact of faith-based health programming on Black youth PA and health outcomes (e.g., improved blood pressure) [49, 50]. Future research is needed to explore the dynamic relationship between small businesses and the types of YPAOs that they support and do not support, especially when prominent negative societal events arise, with a particular focus on YPAOs that may be culturally relevant and appropriate to promote youth PA participation in urban, low-income, predominantly-Black neighborhoods.

This study also identified significant differences in the kinds of support provided by small businesses for YPAOs pre-COVID versus post-COVID. While monetary donations by businesses in support of YPAOs were very similar before (60%) and after (66%) COVID, there was a significant drop in support in the form of goods and services. In addition, volunteerism doubled from 11 to 22% post-COVID despite small businesses having significantly fewer employees post-COVID. It is important to consider these changes in support when addressing future health crises. For example, small businesses could be encouraged to offer non-traditional goods and services in support of YPAOs. During the COVID pandemic, this may have included the provision of free or reduced-cost viral mitigation supplies (e.g., cleaners, masks) by businesses able to provide such goods. Similarly, targeting volunteerism when soliciting small businesses for support could provide a way to keep them involved with YPAOs. Research is needed to determine the extent to which the type of YPAO support from small business impacts YPAO operations and youth PA participation.

A substantial proportion (> 90%) of owners believed a small business should support YPAOs. This was noted whether pre- or post-COVID or if the business was supporting or not supporting YPAOs. Most likely, this resilient belief of the owners contributed to the maintenance of support for YPAOs by small businesses during the pandemic (34% pre-COVID and 27% post-COVID supported YPAOs). Similarly, Hadjielias et al. [51] found that during the COVID pandemic, the resilient qualities of small business owners translated into resilient actions at the small business level. A majority (67%) of owners whose small businesses supported a YPAO post-COVID lived in the same neighborhood, which tripled from pre-COVID. This finding aligns with other research showing that during the COVID-19 pandemic, owners were more likely to live near their small businesses due to the negative economic impact of the pandemic [51]. A 2019 study found that owners who resided in the same communities as their customers were more readily engaged in supporting community health promotion [52]. As owners generally participate in financial decisions (including sponsorships) regarding the business, improving owner buy-in and active involvement with efforts to promote small business support for YPAOs [15, 29, 53]. Owners should also have the option to direct their support to YPAOs near the business. This would be synergistic with small business owners’ tendencies to assist local YPAOs and the positive impact support for local YPAOs has on PA [15, 29–33].

The primary reason (44%) for not supporting YPAOs post-COVID was the inability to locate YPAOs to support. Nearly 44% of post-COVID businesses could not find YPAOs to support versus 6.5% of the pre-COVID businesses. While this is a major barrier, it may be tapered by fostering direct affiliations between small business owners and employees and YPAO leaders and participants as such affiliations predict YPAO support [15]. Strategies YPAOs could follow for soliciting support from small businesses are advised given that very few businesses are ask to support YPAOs. Soliciting support, building affiliations, may help small businesses locate and channel support to YPAOs even when their numbers are substantially reduced. Another mechanism influencing small business support for YPAOs both pre-COVID and post-COVID was “goodwill” (e.g., giving back to the community and affecting youth health). The recognition of the goodwill as a quality of small business sponsorship activities has the potential to attract more customers, especially when expressed in the neighborhood where the business operates [52]. This could further incentivize small business to build and maintain relationships with YPAOs even during a pandemic.

Businesses pre-COVID employed more employees and had much larger budgets for community initiatives and advertising than post-COVID businesses. This likely reflects the adverse economic impact of COVID. A significant decline was observed in the proportion of businesses endorsing non-YPAO community initiatives. For instance, there was about a sevenfold reduction in the percentage of small businesses contributing to non-YPAO initiatives. This stands in contrast to the findings related to YPAO support, where there was no significant change in the percentage of businesses backing YPAOs. It is plausible that as the budgets and operational capacities of small businesses contracted during the pandemic, their ability and willingness to support broader community initiatives waned. Nevertheless, despite these challenges, small businesses, driven by a commitment to the local community, perhaps stemming from a strong belief in fostering goodwill and supporting YPAOs, continued to lend support to initiatives they deemed highly valuable, such as YPAOs. The discovery that a majority of small businesses believe in supporting YPAOs provides some backing for this argument [15, 29].

There are study limitations that should be considered when interpreting the results. First, self-report data were used which is associated with some biases [54]. Although the small business questionnaire employed is reliable, its validity has not been examined. It is recommended that future studies in this area obtain objective information (e.g., policy reviews) to augment data acquired from subjective means. Second, the separate-sample, pretest–posttest design that was utilized is associated with some threats to internal validity [55]. Nonetheless, this design eliminates the effects associated with testing bias (pretest affecting posttest) and the loss-to-follow-up. Additional studies are needed to track changes in small business support for YPAOs over time. Third, data were obtained from residents living in neighborhoods located in a single county, perhaps restricting generalizations to other areas. Lastly, while the focus of this study was on predominantly Black neighborhoods, it would be of interest to extend this line of research to other racial/ethnic localities for comparative purposes.

## Conclusion

The current study provides novel perspectives on changes in small business support for YPAOs after exposure to the COVID-19 pandemic, including marked shifts in the types and reasons for supporting and not supporting YPAOs. The results could be used to inform policy decisions and support mechanisms aimed at helping YPAOs sustain operations during and recover from prominent negative societal events. Such efforts may be enhanced by highlighting aspects found in this study to be related to support for YPAOs by small businesses (e.g., goodwill). Going forward, support for YPAOs by small businesses could benefit by securing business owner buy-in (e.g., by stressing the importance of goodwill, the need for goods and services) and establishing firm relationships between YPAOs and the small business community. Further, developing strategies (e.g., allowing business owners to direct support to specific areas, actively soliciting support) for growing and channeling small business support for YPAOs in underserved areas could potentially help address disparities in youth PA especially disparities resulting from environmental deficiencies. Future research is needed to explore the dynamic relationship between small businesses and the types of YPAOs they do and do not support, especially when prominent negative societal events arise, with a particular focus on YPAOs that are culturally relevant and appropriate to promote youth PA participation in urban, low-income, predominantly Black neighborhoods. In addition, evidence is needed on how small business support for YPAOs can be enhanced and how changes in support affect youth PA participation.

## Consent to Participate

Informed consent was obtained from all participants included in the study.

## Data Availability

The dataset generated during and/or analyzed during the current study is available from author Richard Suminski upon reasonable request.
